# Supplementary material for: Single-molecule visualization of ATP-induced dynamics of the subunit composition of an ECF transporter complex under turnover conditions
Source: Nat Commun. 2025 May 13;16:4448. doi: 10.1038/s41467-025-59674-6 (PMC12075633; doi:10.1038/s41467-025-59674-6)
Supplement: Supplementary file 1 — Supplementary Information [file 41467_2025_59674_MOESM1_ESM.pdf]

Supplementary Figures and Tables

Single-molecule visualization of ATP-induced dynamics of the subunit composition of an ECF transporter complex under turnover conditions

Solène N. Lefebvre, Mark Nijland, Ivan Maslov, Dirk J. Slotboom

S.N.L., M.N., I.M., D.J.S. – Faculty of Science and Engineering, Groningen, Biomolecular Sciences and Biotechnology, Membrane Enzymology Group, University of Groningen, 9747 AG, Groningen, The Netherlands

Correspondence: D.J.S. (d.j.slotboom@rug.nl)

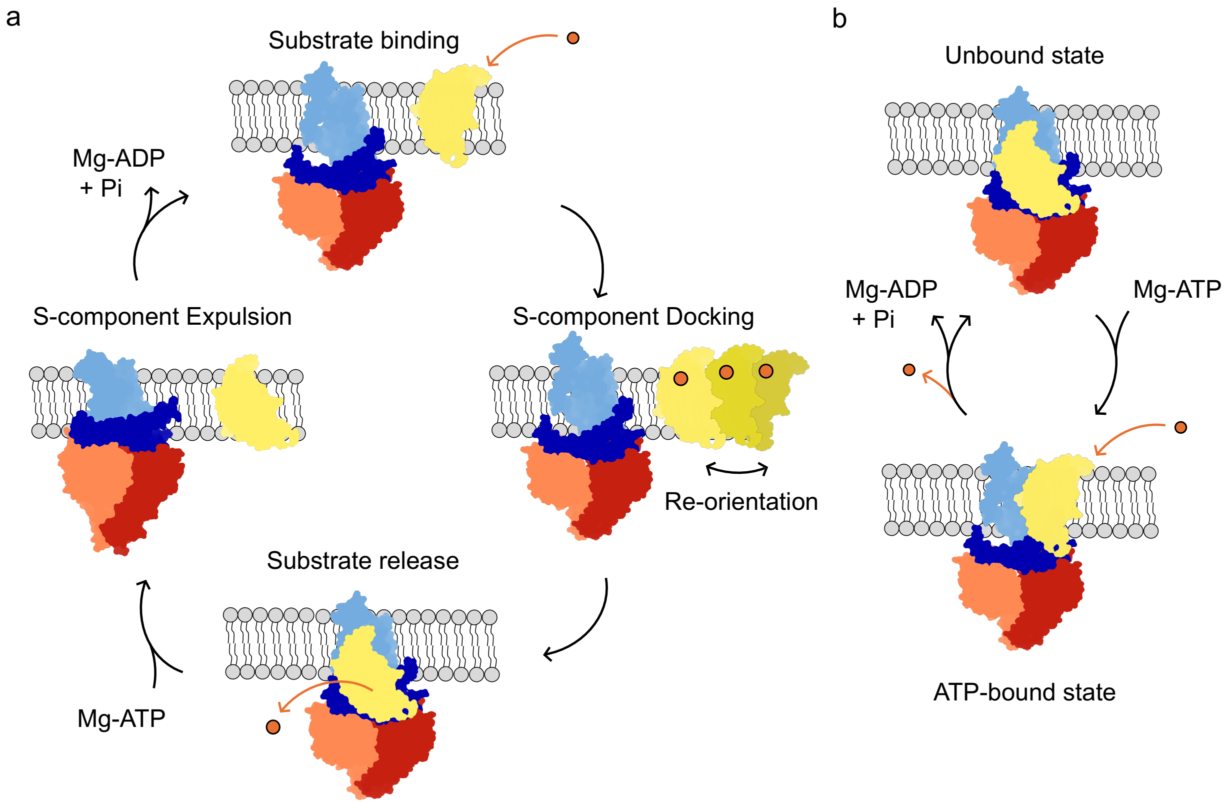

Supplementary Fig. 1 | Schematic representations of two potential transport models. ATPases domains are shown in red and orange, EcfT in blue with the coupling helixes in dark blue and the S-component in yellow. The orange circle represents the substrate. **a,** Thermal ratchet model that includes an ATP-binding step in which the S-component is expelled from the complex after the substrate has been translocated[1]. **b,** Model in which the complex remains assembled, ATP binding precedes the substrate binding and transport steps, and hydrolysis is required for translocation and substrate release on the cytoplasmic side[2].

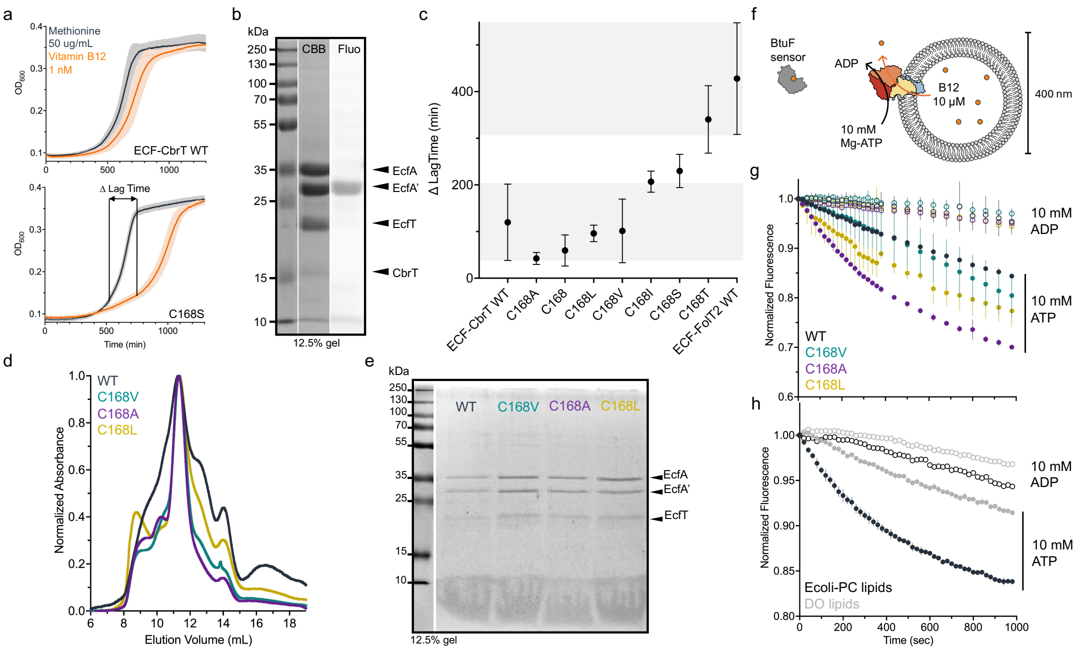

Supplementary Fig. 2 | Optimization of the Cysteine-less background of ECF-CbrT and lipid composition. a, Example of growth assay of the *E. coli* ΔFEC strain expressing ECF-CbrT constructs in minimal M9 medium containing either methionine or vitamin B12. Delay in growth is seen for Δ3C + C168S (all 4 endogenous cysteines substituted with serines) but not for Δ3C (only 3 cysteines substituted) in the vitamin B12 condition indicating lack of expression or not functional transporter when C168 is mutated into serine (average and standard deviation over biological and technical replicates are shown). **b,** SDS-page gel of purified ECF-CbrT Δ3C after fluorescein-maleimide labeling in Coomassie brilliant blue staining (CBB) and fluorescence imaging (Fluo) indicating that C168 on EcfA' is accessible for labeling and should be substituted. **c**, Results of different cysteine substitution growth assays after Gompertz fitting showing the difference of lag time between methionine and vitamin B12 conditions (average and standard deviations over all replicates are shown). WT ECF-CbrT and FolT2 were used as positive and negative controls. C168 substituted in alanine, leucine or valine were most promising with a lower Δ Lag Time. **d,** Characterization of WT and 3 cysteine-less ECF-CbrT constructs with size exclusion chromatography profiles and **e,** SDS-page gel after proteoliposome reconstitution with *E. coli* - PC lipids at a 1:500 protein to lipid ratio. **f,** Schematic of fluorescent transport assay. **g,** Result of fluorescence transport assay showing that alanine is the best substitution for C168, with better transport activity than WT. This assay was done in duplicates with 25 nM of BtuF and data point after 5 min were collected every 60 s. **h,** Transport assay of ECF-CbrT_D77C_Alexa Fluor 647 reconstituted at a protein to lipid ratio of 1:500 either in *E. coli* - PC or DO lipids (both containing 1 % DOPE-biotin) showing that the first composition gives a better transport activity. This transport assay was done in triplicates with 60 nM of BtuF. The DO lipid mix used was DOPE:DOPC:DOPG at 2:1:0.7. In **g** and **h,** empty dots correspond to addition of 10 mM Mg-ADP and filled dots to addition of 10 mM Mg-ATP.

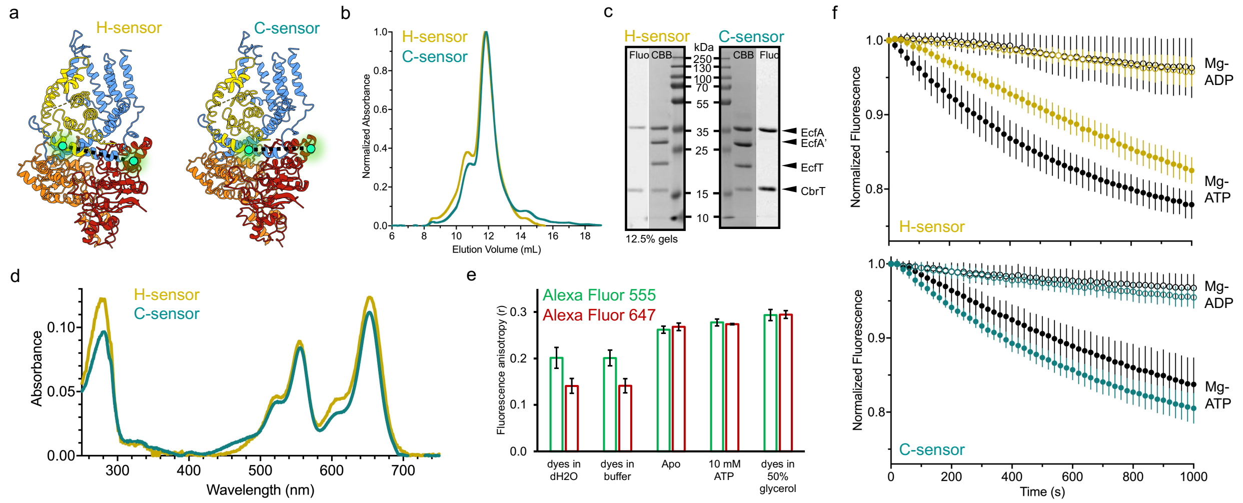

Supplementary Fig. 3 | H- and C-Sensors. a, Structure of ECF-CbrT (pdb code: 6fnp) with CbrT in yellow, ATPases in red and orange and EcfT in blue. C_α atoms of the residues used for the cysteine substitution are shown in green spheres. For the C-sensor, the last residue of CbrT resolved A175 is shown for illustration, while the cysteine is introduced on the unresolved residue A182. **b,** Size exclusion chromatography profiles of the H-sensor (yellow) and C-sensor (green). **c,** SDS-page gels of both sensors showing the 4 subunits in Coomassie brilliant blue staining (CBB) and the two labeled subunits in fluorescence imaging (Fluo). **d,** Absorbance spectra of the H-sensor (yellow) and C-sensor (green) after fluorophore labeling showing the presence of both donor and acceptor dye. Labeling efficiency in this example is 68 % for the H-sensor and 80 % for the C-sensor with the donor dye accounting for slightly more than half of the labeling in both cases (56% donor 44 % acceptor). **e,** Fluorescence anisotropy results of Alexa Fluor 555 and 647 dyes in solution or after labeling on the C-sensor in detergent solution. Addition of ATP to the buffer does not change the anisotropy in the labeled transporter. Anisotropy r-values are given as mean ± SD, with n=3 technical replicates. **f,** Fluorescence transport assay of the H and C-sensors (top and bottom panel respectively) before (black) and after labeling (color) showing that in the case of the H-sensor, the activity is decreased after labeling of the cysteines. Empty dots correspond to the addition of 10 mM Mg-ADP and filled dots to addition of 10 mM Mg-ATP. Average and standard deviations are shown from 5 and 7 repeats for the C-sensor (non-labeled and labeled) and 3 repeats for the Helix sensor (each repeat was done in duplicate or triplicate).

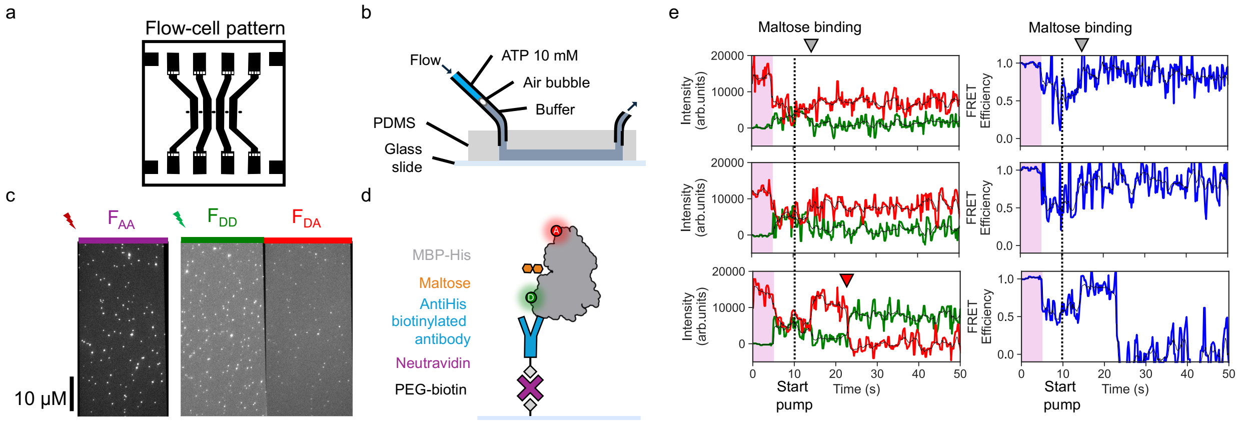

Supplementary Fig. 4 | TIRF recording and maltose binding to MBP experiment. **a,** Pattern of the Flow-cell, containing four 1 mm wide channels, each having at both extremities a bubble trap and a space to punch holes in the PDMS and insert inlet and outlet tubing. Recordings were done in the vertical, middle part of the channels. **b,** Side view of the flow-cell showing a channel with the tubing. In the ATP addition experiment, an air bubble is introduced before the ATP solution to limit diffusion. The bubble is small enough to be blocked by the trap or at the extremity of the tubing and does not go through the channel. **c,** Example of field of view of the acceptor channel during acceptor excitation (F_{AA}) or both donor (F_{DD}) and acceptor (F_{DA}) channel during donor excitation. Images are average intensity projections of 25 frames (5 s). **d,** Schematic of the MBP immobilization. As for proteoliposomes experiments, the flow cells contain a PEG-biotin coated glass slide and neutravidin is incubated in the channels before immobilization of the sample. An additional incubation step is done with anti-his biotinylated antibody to immobilize MBP protein containing a His-tag, labeled at positions T36C and S352C. **e,** Example of smFRET traces from the MBP experiment, donor intensity in green and acceptor in red (black line is the signal after CK filtering). The resulting FRET efficiency is shown in blue in the right panels, the first 5 seconds in the pink shaded area correspond to direct acceptor excitation. Addition of 200 μM maltose was done as shown in panel b, to record the change in FRET efficiency occurring when maltose binds to the MBP. In this recording, the flow starts after 10 seconds of recording and a few seconds later, a change in FRET efficiency is recorded corresponding to the diffusion of maltose in the channel and binding to MBP. The FRET efficiency values of about 0.6 (apo) and 0.8 (bound) are in line with previous in solution recordings[3].

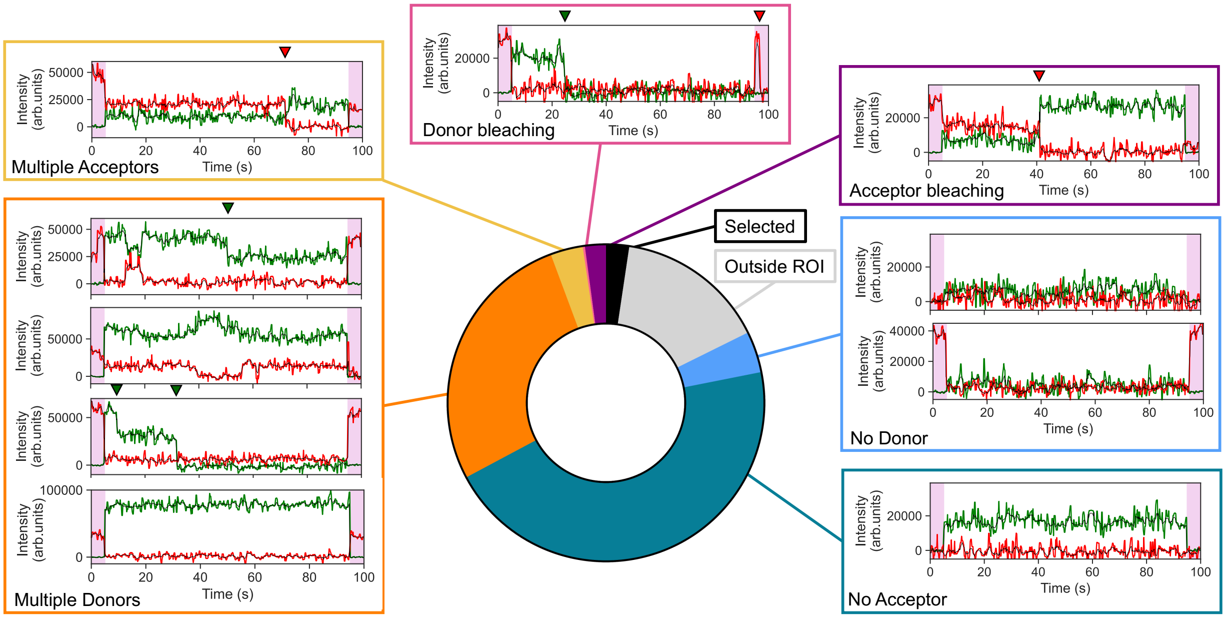

Supplementary Fig. 5 | Selection process of smFRET traces. Example of traces selected out during data processing and their proportion in one representative recording session shown in donut chart. smFRET traces are shown with donor signal in green and acceptor in red (the black line corresponds to the signal after CK filtering). Pink shaded areas in the first and last 5 seconds correspond to direct acceptor excitation. Green and red triangles indicate donor and acceptor photobleaching events respectively. First, to make sure the entirety of the signal coming from one particle is recorded, spots within 10 pixels of the edge of the image in the green channel were selected out (21 pixels from the right side where the beam splitter can be tilted, cf Supplementary Fig. 4c). This corresponds to about 15 % of traces extracted from the movie. Then, traces without donor are removed. Particles are identified from the movies in the donor channel, the intensity threshold is set manually for each movie, voluntarily low to select traces that would have a high-FRET efficiency thus a low donor emission intensity. This process leads to traces with donor emission intensity similar to the background that are selected out if the sum of donor and acceptor intensity during donor excitation is also low (no high-FRET). The proportion of "no donor traces" varies depending on the density of immobilized particles. Similarly, traces without acceptor are removed. Then, traces with intensities higher than the set thresholds are removed as they likely contain multiple donor or acceptor molecules. The third trace in the "multiple donors" panel shows two donor bleaching steps. The trace in the "multiple acceptors" panel shows one bleaching step and the final 5 seconds of direct acceptor excitation is half of the initial acceptor excitation indicating that two acceptors were present and one bleached during the recording. When traces with only one donor and one acceptor remain, a last selection step removes traces where photobleaching occurs. Thanks to the last 5 seconds of direct acceptor excitation, we can differentiate between traces where acceptor bleaching occurs from the traces where dissociation happens.

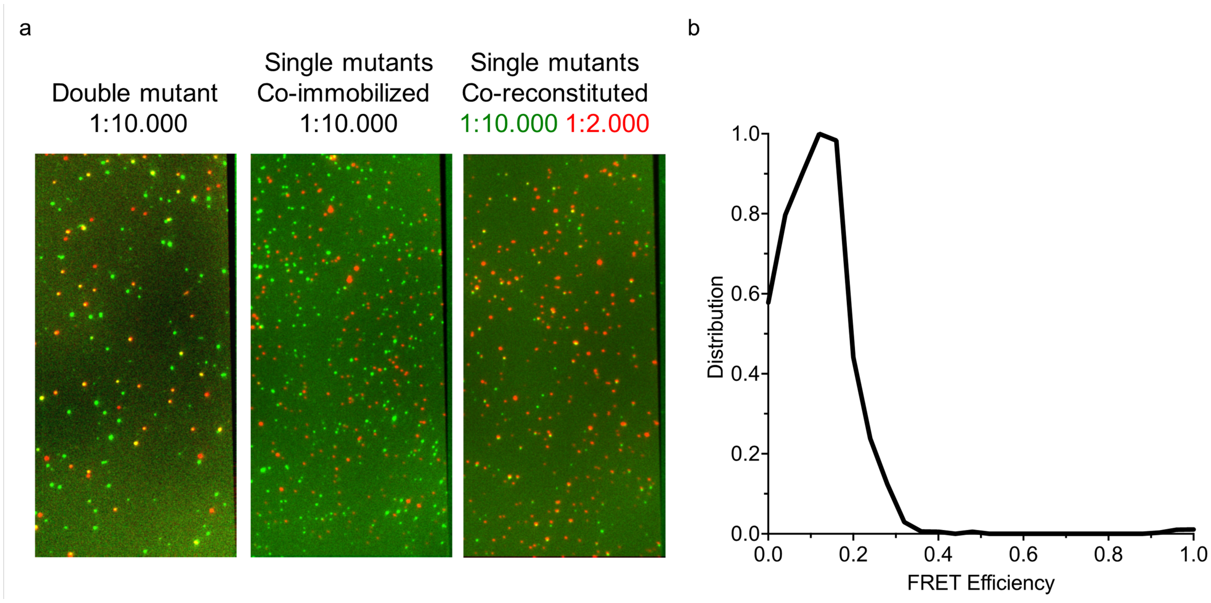

Supplementary Fig. 6 | Colocalization in TIRF recording. a, Example of immobilization of double labeled mutants and single labeled mutants either co-immobilized or co-reconstituted in the indicated protein to lipid ratios. Intensity projections of the first 5 s of acceptor excitation in the acceptor channel (shown in red) and of the first 5 s of donor excitation in the donor channel (shown in green) are overlaid, yellow indicated colocalization of donor and acceptor dyes (mainly seen in double mutant and co-reconstituted sample). **b**, Distribution of FRET Efficiencies from the 8 selected traces in the co-immobilized experiment showing a FRET population similar to the dissociated complex.

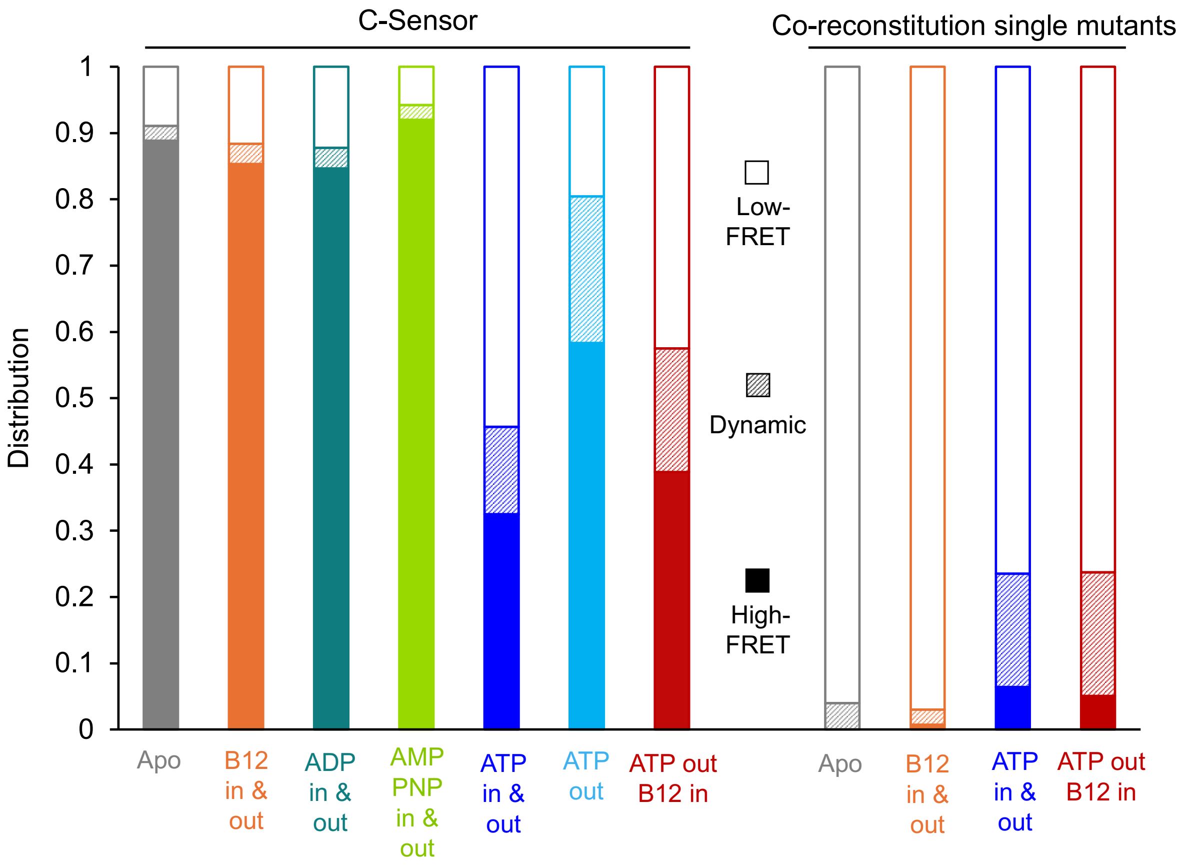

Supplementary Fig. 7 | Distribution of traces in smTIRF recordings. Proportions of High-FRET, low-FRET and dynamic traces are shown for each condition. Co-reconstitution of single mutants is shown as in Figure 3 and the distributions for the double-labeled C-sensor are shown for comparison.

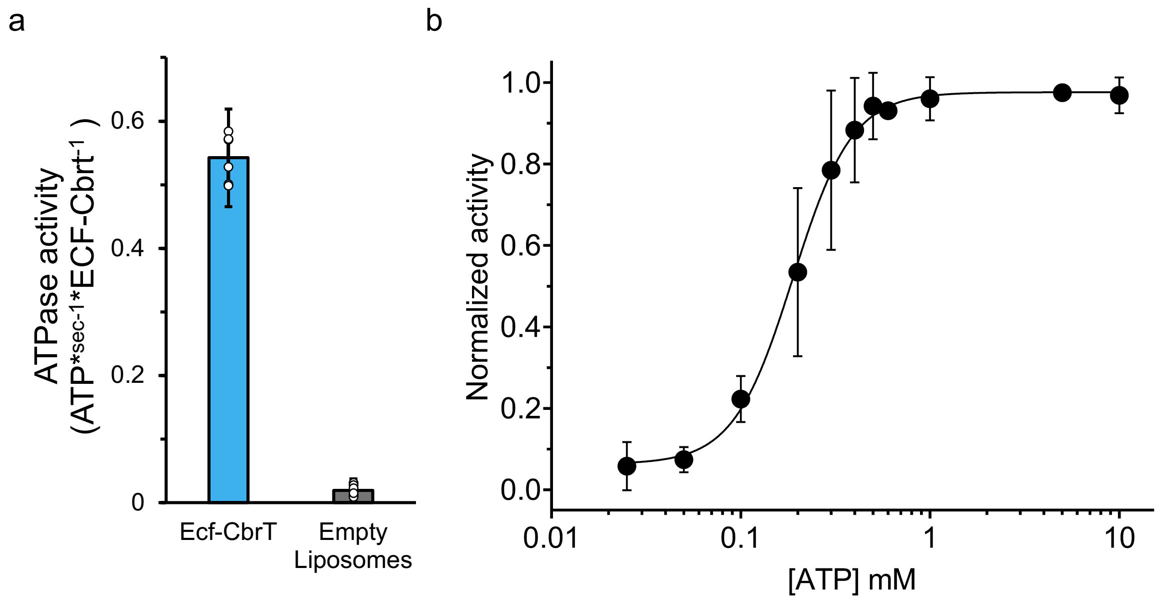

Supplementary Fig. 8 | ATPase activity and apparent K_M for ATP in the vitamin B12 transport assay. a, Results of the coupled enzyme ATPase activity assay on Cysteine-less ECF-CbrT in liposomes showing a hydrolysis rate of around 1 molecule of ATP every 2 seconds per transporter assuming a 100 % reconstitution efficiency and no preferential orientation in the membrane (50 % of transporters with ATPases facing the outside buffer). Average and individual values from biological duplicate and technical triplicates are shown as well as a negative control without transporters. **b,** Results of the transport assay at different ATP concentrations. The apparent K_M was determined on the cysteine-less ECF-CbrT in two independent transport assay measurements (using the vitamin B12 sensor BtuF, similar to the ones in Supplementary Figures 2g,h and 3f) containing duplicates using 13 μM of vitamin B12 in the lumen, 25 nM of BtuF and the indicated concentrations of external Mg-ATP. Linear regression was used to determine the initial transport rates for each ATP concentration in the independent repeats and the normalized averaged values are shown with standard deviation. An apparent K_M for ATP of 0.19 mM was derived by fitting the Hill equation.

Sample	C-Sensor doubly labeled ratio 1:10.000		EcfA_K122C_AlexaFluor647 ratio 1:10.000
Condition	10 μM B12 in 10 mM ATP out		
Recording	7 movies; 3310 extracted traces from green channel		6 movies; 1449 extracted traces from red channel
# Traces	98	415	304

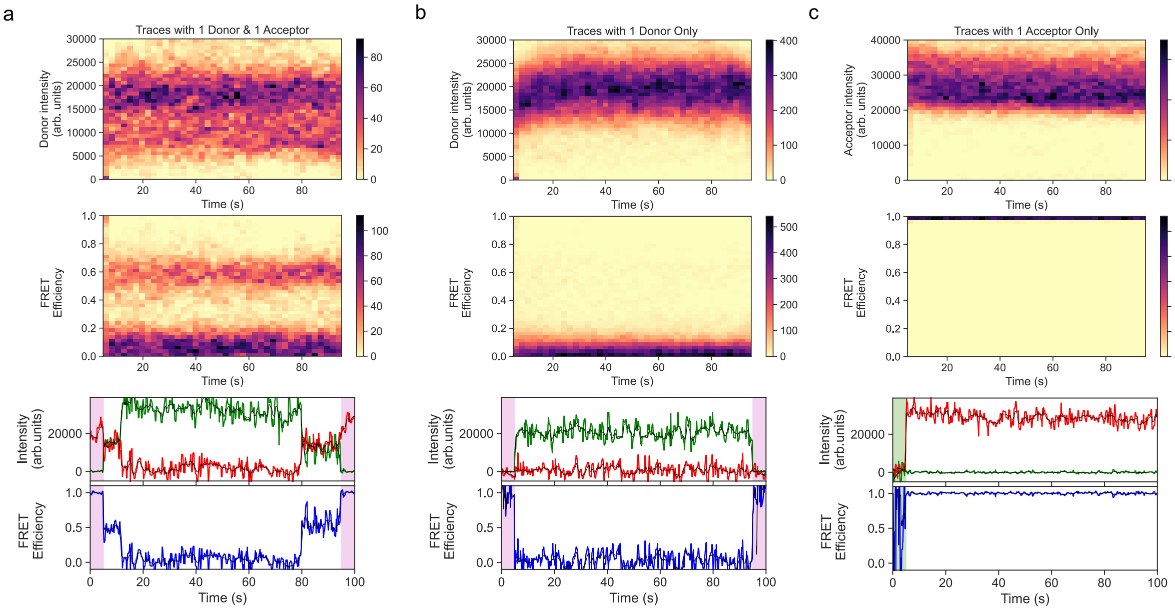

Supplementary Fig. 9 | Overall distribution of FRET efficiencies and intensities in turnover conditions. For traces containing 1 donor and 1 acceptor (**a**); only 1 donor (**b**) and only 1 acceptor (**c**), 2d histograms in top and middle panels show the distribution of fluorescence intensity and FRET efficiency values of the traces from one recording session (number of traces indicated in the table above). Bottom panels show one representative trace for each condition. Purple shaded areas indicate direct acceptor excitation in the first and last 5 seconds for the donor containing traces while for the acceptor only trace, the first 5 seconds (green shaded area) correspond to donor excitation.

a

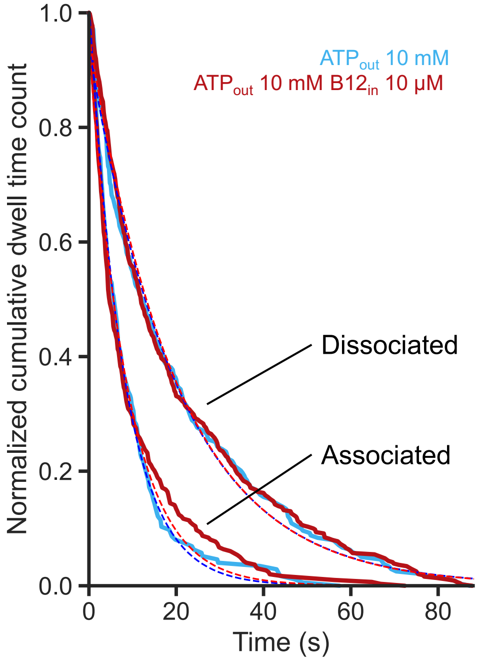

b

	ATP_{out}		ATP_{out} + $B12_{in}$	
	Associated	Dissociated	Associated	Dissociated
# Traces	138		251	
# Dwells	174	239	298	438
A	1.02	0.94	0.95	0.96
τ (s)	7.91	20.37	8.73	20.15
$t_{1/2}$ (s)	5.48	14.12	6.05	13.97

**** (ATPout Associated vs Dissociated)
**** (ATPout + B12in Associated vs Dissociated)
n.s. (Associated: ATPout vs ATPout + B12in)
n.s. (Dissociated: ATPout vs ATPout + B12in)

Supplementary Fig. 10 | Dwell time analysis of the associated and dissociated transporter. a, Distribution of dwell times of associated (High-FRET) or dissociated (Low-FRET) complexes in either ATP_{out} (blue) or ATP_{out} + $B12_{in}$ (red) conditions. One-phase exponential decay fit are shown as dashed lines for each condition **b,** Table recapitulating the number of traces analyzed and dwell times extracted. Parameters of the exponential fitting are shown A is the scaling factor, τ the mean dwell time and $t_{1/2}$ the dwell time half-life. A one-way ANOVA test (right-tailed) was performed using the results of the exponential fitting followed by a Tukey-HSD test (two-tailed). The ATP_{out} and ATP_{out} + $B12_{in}$ conditions were found to be non-significatively different from each other ($p = 0.1446$ and $p = 0.9996$ for associated and dissociated respectively), while the difference between associated and dissociated was significantly different for both conditions ($p < 0.0001$).

Supplementary Table 1 | Bacterial strains and DNA constructs used in this study.

Construct Name	Mutations	Expression Host	
		E. coli **MC1061**	***E. coli*** **ΔFEC**
p2BAD_n10His_ECF-$CbrT_{L.Del}$_WT	-	v	v
p2BAD_n10His _ ECF-$CbrT_{L.Del}$ _Δ3C	EcfT_C252S<br>CbrT_C22S_C86S	-	v
p2BAD_n10His _ ECF-$CbrT_{L.Del}$ _Δ3C_C168A	EcfA'_C168A<br>EcfT_C252S<br>CbrT_C22S_C86S	v	v
p2BAD_n10His _ ECF-$CbrT_{L.Del}$ _Δ3C_C168L	EcfA'_C168L<br>EcfT_C252S<br>CbrT_C22S_C86S	v	v
p2BAD_n10His _ ECF-$CbrT_{L.Del}$ _Δ3C_C168V	EcfA'_C168V<br>EcfT_C252S<br>CbrT_C22S_C86S	v	v
p2BAD_n10His _ ECF-$CbrT_{L.Del}$ _Δ3C_C168I	EcfA'_C168I<br>EcfT_C252S<br>CbrT_C22S_C86S	-	v
p2BAD_n10His _ ECF-$CbrT_{L.Del}$ _Δ3C_C168S	EcfA'_C168S<br>EcfT_C252S<br>CbrT_C22S_C86S	-	v
p2BAD_n10His _ ECF-$CbrT_{L.Del}$ _Δ3C_C168T	EcfA'_C168T<br>EcfT_C252S<br>CbrT_C22S_C86S	-	v
p2BAD_n10His _ ECF-$CbrT_{L.Del}$ _C-Sensor	EcfA_K122C<br>EcfA'_C168A<br>EcfT_C252S<br>CbrT_C22S_C86S_A182C	v	-
p2BAD_n10His _ ECF-$CbrT_{L.Del}$ _K122C	EcfA_K122C<br>EcfA'_C168A<br>EcfT_C252S<br>CbrT_C22S_C86S	v	-
p2BAD_n10His _ ECF-$CbrT_{L.Del}$ _A182C	EcfA'_C168A<br>EcfT_C252S<br>CbrT_C22S_C86S_A182C	v	-
p2BAD_n10His _ ECF-$CbrT_{L.Del}$ _H-Sensor	EcfA_D77C<br>EcfA'_C168A<br>EcfT_C252S<br>CbrT_C22S_C86S_L167C	v	-
p2BAD_n10His _ ECF-$CbrT_{L.Del}$ _D77C	EcfA_D77C<br>EcfA'_C168A<br>EcfT_C252S<br>CbrT_C22S_C86S	v	-
p2BAD_n10His _ Ecf-$FolT2_{L.Del}$_StrepII_WT	-	-	v
pBAD18_n8His_$BtuF_{E.coli}$	D141C _C183S_C259S	v	-

Supplementary Table 2 | Forward (FW) and reverse (RV) primers used in this study.

Primer	DNA sequence 5' to 3'
EcfA_D77C	FW: GGCTTGCACGGTTTGGGAAGTCAG<br>RV: CGTGCAAGCCCCCAGCTTG
EcfA_K122C	FW: GCTTTGCATCGTCGCTCAGGC<br>RV: CGATGCAAAGCATTTCCGGCC
EcfA'_C168A	FW: GATCATCGCTTTAGATGAGCCGGCAGC<br>RV: CATCTAAAGCGATGATCTCCGGCTC
EcfA'_C168L	FW: GAGCCGGAGATCATCCTGTTAGATGAGCCGG<br>RV: CCGGCTCATCTAACAGGATGATCTCCGGCTC
EcfA'_C168V	FW: GCCGGAGATCATCGTTTTAGATGAGCC<br>RV: GGCTCATCTAAAACGATGATCTCCGGC
EcfA'_C168I	FW: GAGCCGGAGATCATCATCTTAGATGAGCCGG<br>RV: CCGGCTCATCTAAGATGATGATCTCCGGCTC
EcfA'_C168S	FW: GAGCCGGAGATCATCAGCTTAGATGAGCCGG<br>RV: CCGGCTCATCTAAGCTGATGATCTCCGGCTC
EcfA'_C168T	FW: GATCATCACTTTAGATGAGCCGGCAG<br>RV: CTAAAGTGATGATCTCCGGCTCATAG
EcfT_C252S	FW: CTACTCCCTGCTGTTGACAATTTTG<br>RV: GCAGGGAGTAGGCTACTGGAATC
CbrT_C22S	FW: GCTGACTGCGATGTCCGTGGTTTTGCGG<br>RV: CCGCAAAACCACGGACATCGCAGTCAGC
CbrT_C86S	FW: GCCTACGCGGCCTCTGCTTTGACCG<br>RV: CGGTCAAAGCAGAGGCCGCGTAGGC
CbrT_L167C	FW: GGTTTGCGGCCTGGACCGGTTTAAAAAG<br>RV: GGCCGCAAACCAATGGCAGATAAAAG
CbrT_A182C	FW: CAAAATTGTTAATGACTCGAGGTTTAAACGG<br>RV: CATTAACAATTTTGCTTCCACCCTGC

Supplementary Table 3 | smFRET TIRF replicates per conditions

Experiment	Condition	# Traces	# Recording sessions	# Movies	# Channels	# Purification batches
Double Mutant	Apo H sensor	54	1	6	2	1
	Apo C sensor	547	16	75	37	4
	10 mM ATP_{out}	624	11	72	22	3
	10 mM $ATP_{in/out}$	289	3	34	6	3
	10 mM ADP	98	1	12	2	1
	5 mM AMPNP	225	1	12	2	1
	10 µM B12	293	2	23	4	2
	10 mM ATP_{out} 10 µM $B12_{in}$	1344	14	115	26	4
Co-Reconstitution	Apo	50	1	12	2	1
	10 µM B12	132	1	12	2	1
	10 mM ATP	234	1	12	2	1
	10 mM ATP_{out} 10 µM $B12_{in}$	59	1	12	2	1
	Co-immobilization	8	1	5	1	1
Dose Response ATP_{out}	25 µM	60	1	6	1	1
	50 µM	138	2	14	2	1
	100 µM	89	2	12	2	1
	200 µM	91	2	10	2	1
	300 µM	164	2	13	2	1
	500 µM	294	5	31	9	2
	1000 µM	171	2	14	2	1
Dose Response ATP_{out} $B12_{in}$	25 µM	32	1	7	1	1
	50 µM	94	2	14	2	1
	100 µM	80	2	13	2	1
	200 µM	89	2	14	2	1
	300 µM	156	2	14	2	1
	500 µM	168	2	13	2	1
	1000 µM	148	2	13	2	1
Addition Mg-ATP / Maltose	10 mM ATP (no B12)	162	9	19	19	2
	10 mM ATP (10 µM $B12_{in}$)	105	6	11	11	3
	200 µM Maltose	93	3	5	5	1

References

1. Thangaratnarajah, C. *et al.* Expulsion mechanism of the substrate-translocating subunit in ECF transporters. *Nat Commun* 14, 4484 (2023).

2. Finkenwirth, F. *et al.* ATP-dependent conformational changes trigger substrate capture and release by an ECF-type biotin transporter. *Journal of Biological Chemistry* 290, 16929–16942 (2015).

3. van den Noort, M., de Boer, M. & Poolman, B. Stability of Ligand-induced Protein Conformation Influences Affinity in Maltose-binding Protein. *J Mol Biol* 433, 167036 (2021).
