# Supplementary material for: Single-molecule visualization of ATP-induced dynamics of the subunit composition of an ECF transporter complex under turnover conditions
Source: Nat Commun. 2025 May 13;16:4448. doi: 10.1038/s41467-025-59674-6 (PMC12075633; doi:10.1038/s41467-025-59674-6)
Supplement: Supplementary file 2 — Reporting Summary [file 41467_2025_59674_MOESM2_ESM.pdf]

Corresponding author(s): D.J. Slotboom

Last updated by author(s): YYYY-MM-DD

Reporting Summary

Nature Portfolio wishes to improve the reproducibility of the work that we publish. This form provides structure for consistency and transparency in reporting. For further information on Nature Portfolio policies, see our Editorial Policies and the Editorial Policy Checklist.

Statistics

For all statistical analyses, confirm that the following items are present in the figure legend, table legend, main text, or Methods section.

n/a	Confirmed	
☐	☒	The exact sample size (*n*) for each experimental group/condition, given as a discrete number and unit of measurement
☐	☒	A statement on whether measurements were taken from distinct samples or whether the same sample was measured repeatedly
☐	☒	The statistical test(s) used AND whether they are one- or two-sided<br>*Only common tests should be described solely by name; describe more complex techniques in the Methods section.*
☒	☐	A description of all covariates tested
☒	☐	A description of any assumptions or corrections, such as tests of normality and adjustment for multiple comparisons
☐	☒	A full description of the statistical parameters including central tendency (e.g. means) or other basic estimates (e.g. regression coefficient) AND variation (e.g. standard deviation) or associated estimates of uncertainty (e.g. confidence intervals)
☐	☒	For null hypothesis testing, the test statistic (e.g. *F*, *t*, *r*) with confidence intervals, effect sizes, degrees of freedom and *P* value noted<br>*Give P values as exact values whenever suitable.*
☒	☐	For Bayesian analysis, information on the choice of priors and Markov chain Monte Carlo settings
☒	☐	For hierarchical and complex designs, identification of the appropriate level for tests and full reporting of outcomes
☒	☐	Estimates of effect sizes (e.g. Cohen's *d*, Pearson's *r*), indicating how they were calculated

Our web collection on statistics for biologists contains articles on many of the points above.

Software and code

Policy information about availability of computer code

Data collection	Growth assay: Gen5 (Biotek); Size exclusion profile: ChromeLab (Biorad); Fluorescence absorbance spectra/anisotropy: Spectra manager (Jasco); Fluorescence-based transport data: SparksControl version 2.3 (TECAN); Coupled Enzyme assay: SoftMax Pro Version 7.1.2 (Molecular devices); Confocal smFRET: SymPhoTime 64 (Picoquant); TIRF smFRET: Micro-manager version 2.0.
Data analysis	smFRET Confocal data processing: Custom python script in JupyterNotebook (Python version 3.6.13, Fretbursts version 0.7.1, phconvert version 0.9.1, NumPy version 1.1.19, pandas version 1.1.5, Matplotlib version 3.3.4, lmfit version 1.0.3); smFRET TIRF data processing: custom plugin in ImageJ, python script in Jupyter Notebook ((Python version 3.12, NumPy version 1.26.4, SciPy 1.13.1, Matplotlib version 3.8.4, Seaborn version 0.13.2) and MASH FRET software (custom scripts and ImageJ plugin are provided on Github at https://github.com/MembraneEnzymology/). Data visualization and statistical tests: Prism version 10.0.2 (Graphpad Inc.), ChimeraX version 1.4 and customed python script (Python version 3.12, NumPy version 1.26.4, Matplotlib version 3.8.4, Seaborn version 0.13.2).

For manuscripts utilizing custom algorithms or software that are central to the research but not yet described in published literature, software must be made available to editors and reviewers. We strongly encourage code deposition in a community repository (e.g. GitHub). See the Nature Portfolio guidelines for submitting code & software for further information.

Data

Policy information about availability of data

All manuscripts must include a data availability statement. This statement should provide the following information, where applicable:

- Accession codes, unique identifiers, or web links for publicly available datasets
- A description of any restrictions on data availability
- For clinical datasets or third party data, please ensure that the statement adheres to our policy

The data supporting this study are available from the corresponding authors upon request. The previously solved structure of Ecf-CbrT is available through the ProteinData Bank (PDB) under the accession code 6FNP. Protein sequences used in this study are available through UniProt under the accession codes P37028 for BtuF, Q1GBJ0 for EcfA, Q1GBI9 for EcfA', Q1GBI8 for EcfT, Q1G292 for FolT2, and Q1G7W0 for CbrT. The source data underlying Figures and Supplementary Figures are provided as Source Data file.

Research involving human participants, their data, or biological material

Policy information about studies with human participants or human data. See also policy information about sex, gender (identity/presentation), and sexual orientation and race, ethnicity and racism.

Reporting on sex and gender	NA
Reporting on race, ethnicity, or other socially relevant groupings	NA
Population characteristics	NA
Recruitment	NA
Ethics oversight	NA

Note that full information on the approval of the study protocol must also be provided in the manuscript.

Field-specific reporting

Please select the one below that is the best fit for your research. If you are not sure, read the appropriate sections before making your selection.

☒ Life sciences ☐ Behavioural & social sciences ☐ Ecological, evolutionary & environmental sciences

For a reference copy of the document with all sections, see nature.com/documents/nr-reporting-summary-flat.pdf

Life sciences study design

All studies must disclose on these points even when the disclosure is negative.

Sample size	No sample size determination was performed. Experiments were performed multiple times with similar results and further inclusion of data did not change the results.
Data exclusions	No data was excluded.
Replication	Experiments were repeated multiple times and the number of replicates and errors are indicated in the manuscript. All attempts at replication were successful.
Randomization	Samples and organisms were not allocated into experimental groups, thus randomization is not relevant for our study. However, single colonies for growth assays were picked randomly.
Blinding	There was no group allocation during data collection and/or analysis making blinding not necessary.

Reporting for specific materials, systems and methods

We require information from authors about some types of materials, experimental systems and methods used in many studies. Here, indicate whether each material, system or method listed is relevant to your study. If you are not sure if a list item applies to your research, read the appropriate section before selecting a response.

Materials & experimental systems

n/a	Involved in the study
x	Antibodies
x	Eukaryotic cell lines
x	Palaeontology and archaeology
x	Animals and other organisms
x	Clinical data
x	Dual use research of concern
x	Plants

Methods

n/a	Involved in the study
x	ChIP-seq
x	Flow cytometry
x	MRI-based neuroimaging

Plants

Seed stocks	NA
Novel plant genotypes	NA
Authentication	NA
